# Deficits in emotion processing in Alzheimer’s disease: a systematic review

**DOI:** 10.1590/1980-57642021dn15-030003

**Published:** 2021

**Authors:** Rogeria Cristina Rangel da Silva, Raquel Luíza Santos de Carvalho, Marcia Cristina Nascimento Dourado

**Affiliations:** 1 Center for Alzheimer’s Disease, Institute of Psychiatry, Universidade Federal do Rio de Janeiro – Rio de Janeiro, RJ, Brazil.; 2 Universidade Grande Rio – Rio de Janeiro, RJ, Brazil.

**Keywords:** Alzheimer’s disease, cognition, emotion, doença de Alzheimer, cognição, emoção

## Abstract

Emotional processing involves the ability of the individual to infer emotional information. There is no consensus about how Alzheimer’s disease (AD) affects emotional processing. Objective: Our aim is to systematically review the impact of AD on emotion processing.

**Methods:**

We conducted a search based on the Preferred Reporting Items for Systematic Reviews and Meta-Analyses (PRISMA). The literature search was performed using the electronic databases MEDLINE (PubMed) and Science Citation Index (Institute for Scientific Information [ISI]). The following descriptors were used in the review process: emotion or emotional processing, cognition or cognitive functions, and Alzheimer disease or Alzheimer’s disease. This systematic review was recorded in the International Prospective Register of Systematic Reviews (PROSPERO) under the number CRD42018115891.

**Results:**

We identified 425 articles, 19 of which met our criteria. Visual emotional stimuli were the most used among studies. Most studies used tasks of emotional naming, discrimination, identification, and correspondence. The results were contradictory. Many studies reported that individuals with AD were impaired on emotional perception tasks, while other results reported preserved skills. The relationship between emotional processing and cognition is also unclear. Some studies suggested that general cognitive performance affects performance in emotional perception tasks among people with AD, but other studies have shown deficits in recognizing emotion, regardless of cognitive performance.

**Conclusions:**

Studies are scarce, present contradictory results, and report impairment in emotional processing in relation to cognition. Moreover, the analyses of the correlation between emotion processing and cognitive functioning failed to reveal clear relationships.

## RESUMO

O processamento emocional é a habilidade de indivíduos inferirem informação emocional. Não há consenso sobre como a doença de Alzheimer (DA) afeta o processamento emocional.

**Objetivo:**

Este artigo visa revisar sistematicamente o impacto da DA no processamento emocional.

**Métodos:**

Realizamos uma pesquisa com base no *Preferred Reporting Items for Systematic Reviews and Meta-Analyses* (PRISMA). A pesquisa bibliográfica foi realizada nas bases de dados eletrônicas MEDLINE (Pubmed) e *Science Citation Index* (ISI). Os seguintes descritores foram utilizados no processo de revisão: processamento emocional ou emoção, cognição ou funções cognitivas e doença de Alzheimer. A revisão sistemática foi registrada no Registro Prospectivo Internacional de Revisões Sistemáticas (PROSPERO), sob o número CRD42018115891.

**Resultados:**

Foram identificados 425 artigos, 19 dos quais atendiam aos critérios de inclusão. Os estímulos visuais emocionais foram os mais utilizados entre os estudos. A maioria dos estudos usou tarefas de nomeação emocional, discriminação, identificação e correspondência. Os resultados foram contraditórios. Muitos estudos relataram que indivíduos com DA apresentavam comprometimento em tarefas de percepção emocional, outros resultados apontam para habilidades preservadas. A relação entre o processamento emocional e a cognição também não está clara. Alguns estudos sugerem que o desempenho cognitivo geral afeta o desempenho nas tarefas de percepção emocional na DA, outros estudos demonstraram déficits no reconhecimento da emoção, independentemente do desempenho cognitivo.

**Conclusões:**

Os estudos são escassos, apresentam resultados contraditórios e relatam comprometimento no processamento emocional em relação à cognição. Além disso, análises de correlação entre processamento emocional e funcionamento cognitivo não conseguiram revelar relações claras.

## INTRODUCTION

Emotion processing refers to the cognitive processes involved in the capacity of understanding other people’s and/or one’s own emotional state.^1^ Everyday emotions involve a blending of various bottom-up processing of encounters with emotional stimuli along with top-down conceptual knowledge, memories, and linguistic representations.^2^ Emotional perception may be impaired in healthy aging.^3^ ^,^ ^4^ ^,^ ^5^ Therefore, deficits in emotion processing can be a prominent clinical symptom after acute brain damage, such as traumatic brain injury or stroke, and can be a core feature of the early stages of some neurodegenerative disorders, such as behavioral-variant frontotemporal dementia.^6^ ^,^ ^7^ ^,^ ^8^ ^,^ ^9^

In the mild stages of Alzheimer’s disease (AD), emotion processing impairments might be relatively subtle and harder to detect.^8^ People with AD tend to show decreased emotion processing with impaired identification, labeling, matching, and discrimination of emotions.^6^ ^,^ ^7^ ^,^ ^10^ ^,^ ^11^ Emotion processing has been examined through photos of faces expressing six basic emotions, namely, happiness, sadness, fear, anger, disgust, and surprise.^12^ Other instruments (i.e., emotional dynamic stimuli such as video clips and movie excerpts) may offer additional information about people with the emotional state of AD. There is some evidence that decreased emotion processing among people with AD may be explained by the structural and functional changes within the frontal and temporal neuronal circuits.^13^ ^,^ ^14^ AD pathology, particularly neurofibrillary tangles, initially affects the medial temporal lobe structures, including the entorhinal cortex, hippocampus, and amygdala.^15^ ^,^ ^16^ Various studies highlight the essential role of the amygdala in the emotion processing,^17^ ^,^ ^18^ ^,^ ^19^ ^,^ ^20^ which is supported by neuroimaging studies that show the alterations in the limbic system in people with AD.^21^ ^,^ ^22^ ^,^ ^23^ ^,^ ^24^ Studies also suggest that people with AD showed impaired emotion recognition, especially for facial expressions.^25^ ^,^ ^26^ ^,^ ^27^ ^,^ ^28^ However, others have shown that decoding of emotions is comparable in healthy older adults and people with AD.^26^ In addition, these difficulties do not seem to depend on disease severity.^29^ ^,^ ^30^

Specific emotions may vary in their vulnerability to AD pathology. Recent studies indicate that the capacity to identify happiness (i.e., positive emotions) is preserved in AD, while decoding negative emotions seems impaired.^16^ ^,^ ^27^ ^,^ ^31^ The identification of disgust may be preserved,^25^ while decoding fear is impaired.^6^ ^,^ ^11^ ^,^ ^25^ ^,^ ^27^ ^,^ ^32^ ^,^ ^33^ ^,^ ^34^ There are controversies about people with AD ability to recognizesadness, anger, and surprise^11^ ^,^ ^27^ ^,^ ^34^ ^,^ ^35^. Therefore, we may assume that in AD, the emotion processing is differently affected, and that the identification of some specific emotions may be impacted, while others are not.

Although it was not consistently demonstrated throughout the literature,^6^ ^,^ ^10^ ^,^ ^36^ some authors also suggested that the observed impairment of emotion processing tasks in AD is not necessarily related to impaired emotional decoding abilities, but may, in fact, occur secondary to generalized global cognitive decline.^1^ ^,^ ^13^ ^,^ ^34^ ^,^ ^37^ ^,^ ^38^ However, other authors did not obtain the same results and concluded that people with AD may present impaired emotional recognition *per se*. Thus, given the current controversial results about the impairment of emotion processing in AD, we hypothesized that people with AD may have an impaired capacity of processing emotional information even in the mild stages of the disease. Deficits in emotion processing have been associated with negative caregiver outcomes^39^ and functional disability^8^ in AD. Therefore, in this systematic review, we aimed to better understand the extent to which emotion processing is impaired in people with AD.

## METHODS

This systematic review has been performed according to the Preferred Reporting Items for Systematic Reviews and Meta-Analyses (PRISMA). We searched the Thomson Reuters Web of Science (Institute for Scientific Information – ISI) and MEDLINE (PubMed), including articles published from January 1, 2009, and November 11, 2019, in English, which presented studies with cross-sectional or longitudinal designs about the relationship between emotion processing and cognition among people with AD.

We applied the key words “emotion,” “emotional processing,” “cognition,” and “Alzheimer’s disease” in different combinations for each database. For MEDLINE (PubMed), we applied the following combinations: (emotional processing OR emotional [MeSH Terms] OR emotion [MeSH Terms]) AND (alzheimer disease OR alzheimer’s disease [MeSH Terms]) AND (cognition AND cognitive functions [MeSH Terms]), with the following filters: Humans, English, and Publication Date from 2009/01/01 to 2019/11/01.

For the identification of articles in the Thomson Reuters Web of Science (ISI), we applied the following search strategy: #1 (TS=(alzheimer disease OR alzheimer’s disease) #2 (TS=(emotion OR emotional processing) #3 (TS=(cognition OR cognitive functions OR cognitive functioning) #3 AND #2 AND #. We also applied the following filters: Language: English; types of documents: articles; and date range: 2009–2019. The exclusion criteria included systematic reviews, meta-analysis, articles with no casuistic, and case reports or studies about emotion processing and cognition in other diseases. Initial article-screening was performed by two authors *via* abstract review. We excluded articles that clearly did not fulfill the inclusion criteria, and we maintained the ones that were possibly eligible. When there was not a consensus among the two authors, the article remained as potentially eligible and was included in the next assessment of eligibility. Disagreement between both authors was solved by an independent author.

**Figure 1.**
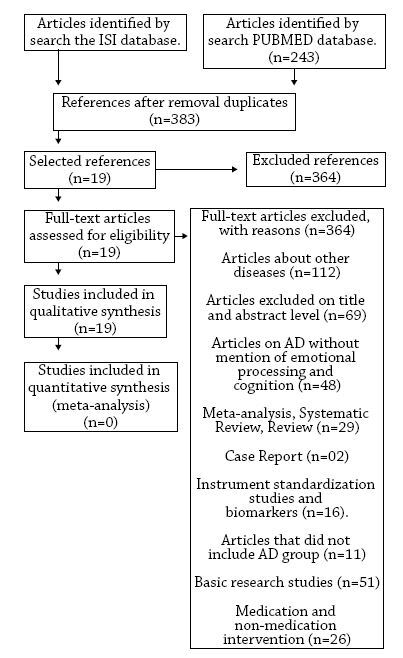
Flow-chart of article selection.

The selected articles from the first phase were read by two independent authors to adjust eligibility. In this phase, the main exclusion criteria for each study were registered in the PRISMA flowchart (Figure 1) to compose the flow of article selection.

To maintain the standard suggested by the PRISMA method, this systematic review was registered in the International Prospective Register of Ongoing Systematic Reviews (PROSPERO) as CRD42018115891.

All the included articles were submitted to the Mixed Methods Appraisal Tool version 2018,^40^ a tool of critical assessment that evaluates the quality of empirical studies.

## RESULTS

We found 425 articles, 182 on the ISI database and 243 on PubMed/MEDLINE. After excluding the cross-references (n=42), we selected 383 articles. The excluded articles (n=364) were selected and analyzed according to the eligibility criteria. The exclusion flowchart of selected articles is described in Figure 1.

We included the complete version of the remaining 19 articles. The selected articles were fully accessed and independently organized as shown in Table 1 that contained the following information: author/year, design, sample, operationalization of AD diagnosis, objective, emotional processing evaluation, cognitive evaluation, and quality assessment.

**Table 1.** Characteristics of the studies.

Authors, year	Design	Sample	AD diagnosis	Objective	Emotional processing evaluation	Cognitive evaluation	Quality assessment
Bourgin, 2018^41^	Cross-sectional	AD 16 HOAs 25	NINCDS-ADRDA Imaging exams	To determine whether early emotional attention mechanisms are affected by AD.	An eye-tracking technique during a simple but time-constrained cognitive task.	MMSE BDI	*****
Daley, 2018^39^	Cross-sectional	AD=28 OCs 30	NINCDS-ADRDA	To investigate the relationship between emotional perception abilities in AD participants and CG well-being.	Advanced Clinical Solutions Social Perception subtest (ACS-SP)	Montreal Cognitive Assessment (MoCA).	*****
Dourado, 2019^56^	Cross-sectional	52 participants AD (mild n=29; moderate n=23)	DSM-IV-TR Imaging exams	To compare facial expression recognition in this AD sample and to identify which factors were associated with the impairment of the ability according to disease severity	The protocol FACES	Assessment Scale– Cognitive Subscale (ADAS-Cog), MMSE, Wechsler Digit Span Test, TMT And Semantic fluency tests	*****
Duclos, 2018^42^	Cross-sectional	n=60 AD 20 HOS 20 and HYS 20	NINCDS-ADRDA	To assess both decoding and reasoning processes in AD, as well as the effect of context on emotion attribution	Peter and Mary emotion tasks Battery	MMSE	*****
Giffard, 2009^43^	Cross-sectional	AD 26 CG 26	NINCDS-ADRDA	To introduce and control the emotional nature of concepts in order to conduct a more ecological investigation of semantic memory in AD using a semantic and affective priming paradigm.	The lexical decision task was composed of 270 pairs of stimuli.	MDRS	****
Kalenzaga, 2013^44^	Cross-sectional	AD 22 CG 21	NINCDS-ADRDA	To explore the impairment in patients with AD working self with regard to the affective dimension of AD.	Remember/Know/Guess paradigm following encoding of emotional and neutral words.	MDRS MMSE	****
Kalenzaga, 2013^45^	Cross-sectional	n=40 AD 22 CG 18	NINCDS-ADRDA	To investigate the effect of emotional words encoded with reference to the self on the state of consciousness associated with memory retrieval in AD.	Remember/Know/Guess paradigm following encoding of emotional and neutral words.	MMSE MDRS	****
Kalenzaga, 2014^46^	Cross-sectional	n=33 AD 18 CG 15	NINCDS-ADRDA	To investigate emotional memory enhancement (EME) in AD. And in exploring which memory process (i.e., recollection or familiarity) could be improved by emotional information in the course of the disease.	Remember/Know/Guess paradigm following encoding of emotional and neutral words.	MDRS	****
Kalenzaga, 2016^47^	Cross-sectional	n=155 YA 38 OA 39 VOA 37 AD 41	NINCDS-ADRDA	To introduce some elements concerning positivity effect in aging	Emotional valence rating using an 8-point scale (1 highly negative, 8 highly positive).	MMSE	****
Laisney, 2013^48^	Cross-sectional	16 AD 15 CG	NINCDS-ADRDA	To investigate changes in the cognitive and affective dimensions of ToM in AD	Affective ToM was assessed *via* a modified version of the Reading the Mind in the Eyes test	Cognitive ToM was assessed by means of preference judgment (PJ) and FB tasks.	*****
Maki, 2013^49^	Cross-sectional	n=54 AD 12 ANC 17 YNC 25	NINCDS-ADRDA	To examine whether recognition of positive facial expressions is preserved in patients with AD	DB99 (Advanced Telecommunications Research Institute International)	CDR	*****
Mograbi, 2012^50^	Cross-sectional	n=43 AD 22 CG 21	DSM-IV-TR	To explore emotional reactivity in mild to moderate AD using film material, investigating the influence of dementia-related material and awareness of condition.	The rating of facial expressions was made with EMFACS, a selective system based on the Facial Action Coding System (FACS).	MMSE, CERAD	****
Mograbi, 2012^51^	Cross-sectional	AD 23 CG 21	DSM-IV-TR	To investigate emotional reactions to success or failure in tasks despite unawareness of performance in AD.	The rating of facial expressions was made with EMFACS, a selective system based on the Facial Action Coding System (FACS). and Mann–Whitney *U*-test.	MMSE, CERAD	*****
Monti, 2010^52^	Cross-sectional	PRAD 19 HEA 15 HYA 22	NINCDS-ADRDA	To evaluate the efficacy of non-emotional and emotional conflict adaptation mechanisms in healthy young, healthy elderly, and PRAD.	Presentation software Pictures of Facial Affect FACS	CDR	***
Sapey, 2015^53^	Cross-sectional	n=78 AD 39 HC 39	NINCDS-ADRDA	To better specify the early emotion recognition deficits at mild stages of AD and to disentangle their neuroanatomical correlates.	Facial emotional expression recognition task.	MMSE, CDR	****
Sava, 2017^54^	Cross-sectional	n=63 AD 17 HO 21 HYs 25 n=60 AD 18 HO 21 HYs 21	NINCDS-ADRDA Brain morphology imaging	To compare the memory performance of healthy young and older participants and patients with AD for faces with positive, neutral, and negative emotional expressions in tasks in which memory performance could not be enhanced by familiarity.	Montreal Set of Facial Displays of Emotion	MMSE	*****
Seid, 2012^57^	Cross-sectional	AD 47	NINCDS-ADRDA	To explore the determinants of emotional facial expression in AD and to address the impact of cognitive and neuropsychiatric symptoms, which were considered as potential moderating variables.	International affective picture system (IAPS). The rating of facial expressions was made with EMFACS, a selective system based on the Facial Action Coding System (FACS).	MMSE, GDS, NPI AES	****
Torres, 2015^58^	Longitudinal	AD 30	DSM-IV-TR And NINCDS-ADRDA	To investigate the patterns of change in the ability to recognize emotions in facial expressions among people with mild AD and to explore the sociodemographic and clinical factors that may influence facial emotion recognition in AD over time	The protocol FACES	CDR MMSE	*****
Werheid, 2011^55^	Cross-sectional	AD 18 CG 18	Not reported	To compare patients with mild AD and age-matched healthy adults with respect to performance in a recognition task involving positive, negative, and neutral faces.	Benton Facial Recognition Test PANAS	MEEM CERAD	****

AD: Alzheimer’s disease; (****) Quality assessment, ADAS-Cog: Assessment Scale–Cognitive Subscale; AES: Apathy Evaluation Scale; ANC: aged normal controls; BDI: Beck Depression Inventory; CDR: Clinical Dementia Rating scale; CERAD: Consortium to establish a registry for Alzheimer’s disease; CG: control group; DSM-IV-TR: *Diagnostic and Statistical Manual of Mental Disorders*, Fourth Edition; FB: false belief; GDS: Global Deterioration Scale; HC: healthy control; HEA: healthy elderly adults; HO: healthy older; HOAs: healthy older adults; HOS: healthy older individuals; HYA: healthy young adults; HYs: healthy young individuals; IAPS: international affective picture system; MDRS: Mattis Dementia Rating Scale; MMSE: Mini-Mental State Examination; MoCA: Montreal Cognitive Assessment; NINCDS-ADRDA: National Institute of Neurological and Communicative Diseases and Stroke/Alzheimer’s Disease and Related Disorders Association; NPI: Neuropsychiatric Inventory; OA: older adults; OCs: older controls; PANAS: Positive and Negative Affect Schedule; PJ: preference judgment; PRAD: probable Alzheimer’s disease; TMT: Trail Making Test; ToM: Theory of Mind; VOA: very old adults; YA: young adults; YNC: young normal controls.

### Sample

From the 19 articles analyzed in this review, 16 studies compared AD with a control group.^39^ ^,^ ^41^ ^,^ ^42^ ^,^ ^43^ ^,^ ^44^ ^,^ ^45^ ^,^ ^46^ ^,^ ^47^ ^,^ ^48^ ^,^ ^49^ ^,^ ^50^ ^,^ ^51^ ^,^ ^52^ ^,^ ^53^ ^,^ ^54^ ^,^ ^55^ One study assessed people with AD in mild and moderate stages.^56^ Seidl et al.^57^ assessed one group of 47 individuals with AD, but they did not specify the stage of disease severity. In Torres et al.,^58^ the comparison was performed in two different moments of people with mild AD.

### Operationalization of Alzheimer’s disease diagnosis

Diagnoses of possible or probable AD in 13 studies were made according to the criteria of the National Institute of Neurological and Communicative Disorders and Stroke and the Alzheimer’s Disease and Related Disorders Association (NINCDS/ADRDA)^39^ ^,^ ^41^ ^,^ ^42^ ^,^ ^43^ ^,^ ^44^ ^,^ ^45^ ^,^ ^46^ ^,^ ^47^ ^,^ ^48^ ^,^ ^49^ ^,^ ^52^ ^,^ ^53^ ^,^ ^54^ ^,^ ^57^ or based on the *Diagnostic and Statistical Manual of Mental Disorders*, Fourth Edition (DSM-IV) guidelines.^50^ ^,^ ^51^ ^,^ ^56^ ^,^ ^58^ Computed tomography and/or magnetic resonance imaging examinations were occasionally used to support the diagnosis.^41^ ^,^ ^48^ ^,^ ^53^ ^,^ ^54^ ^,^ ^56^ ^,^ ^58^ The specific diagnostic criteria used to identify possible or probable AD were unclear in one study.^55^

### Degree of severity in Alzheimer’s disease

Of the 19 studies, 8 studies analyzed the impairment in patients with AD at mild and moderate levels,^42^ ^,^ ^43^ ^,^ ^46^ ^,^ ^47^ ^,^ ^48^ ^,^ ^50^ ^,^ ^51^ ^,^ ^56^ 5 studies evaluated individuals with mild AD,^49^ ^,^ ^53^ ^,^ ^54^ ^,^ ^55^ ^,^ ^58^ and 6 studies did not measure staging or the severity of the disease in the participants of the AD group.^39^ ^,^ ^41^ ^,^ ^44^ ^,^ ^45^ ^,^ ^52^ ^,^ ^57^

## ASSESSMENT CRITERIA

Tasks with various visual stimuli were most frequently applied. In 14 studies, images or drawings were applied.^39^ ^,^ ^41^ ^,^ ^42^ ^,^ ^48^ ^,^ ^49^ ^,^ ^50^ ^,^ ^51^ ^,^ ^52^ ^,^ ^53^ ^,^ ^54^ ^,^ ^55^ ^,^ ^56^ ^,^ ^57^ ^,^ ^58^ Images of facial expressions in black and white were applied in three studies.^52^ ^,^ ^54^ ^,^ ^55^ Color images of natural or manufactured objects were applied in three studies.^41^ ^,^ ^48^ ^,^ ^49^ Two studies applied movies^42^ ^,^ ^50^ using movie excerpts and a short black-and-white video. Finally, one study^51^ also applied two computerized paradigms of success–failure manipulation.

The five remaining studies used a word list as a stimulus.^43^ ^,^ ^44^ ^,^ ^45^ ^,^ ^46^ ^,^ ^47^ The words in word lists were classified as positive, negative, and neutral,^43^ ^,^ ^47^ as well as adjective lists related to personality traits^44^ ^,^ ^45^ and noun lists standardized by Bonin et al.^46^ ^,^ ^59^ in which the words were classified according to emotional state using a 5-point scale from 1 as “very unpleasant” to 5 as “very pleasant.”

From the 19 studies, 9 studies analyzed the following basic emotions: happiness, surprise, anger, sadness, fear, and disgust. Four studies included facial expressions of fear,^39^ ^,^ ^49^ ^,^ ^52^ ^,^ ^53^ and the facial expression of surprise was investigated in five studies.^39^ ^,^ ^42^ ^,^ ^49^ ^,^ ^56^ ^,^ ^58^ Only three studies analyzed the recognition of facial expressions of disgust,^39^ ^,^ ^49^ ^,^ ^53^ and four studies^39^ ^,^ ^53^ ^,^ ^54^ ^,^ ^55^ also included neutral expressions. Dynamic emotional stimuli such as video clips and short movie excerpts were used to assess the emotional processing in two studies.^42^ ^,^ ^50^

## TASKS FOR ASSESSMENT

The assessment tasks were frequently divided into complementing stages according to the level of required cognitive effort. These stages were frequently identified in the studies as emotion identification, discrimination, selection, and correspondence of emotions. These procedures assess various cognitive competences and are considered the first step of emotion processing impairment analysis. Table 2 shows a summary of the specific tasks used in each initial study and includes the main results.

**Table 2.** Tasks and stimuli for the evaluation of emotion processing.

S. No.	Author, Year	Sample	Stimuli and emotions	Tasks	Control tasks	Results
01	Bourgin, 2018^41^	AD=16 HOAs=25	The stimuli were 96 color images of natural or manufactured objects, presented against a white background at a visual angle of 9×9.	Saccadic task involving both prosaccades and antisaccades.	An eye-tracking technique during a simple but time-constrained cognitive task	The results suggest that early emotional attention is indeed impaired in AD
02	Daley, 2018^39^	AD=28 CG = 28 OCs=30	The Advanced Clinical Solutions Social Perception subtest (ACS-SP) to measure emotional perception abilities). Happy, sad, angry, disgusted, afraid, surprised, or neutral.	The Affect Naming task, the Prosody-Face Matching task, and the Prosody-Pair Matching task.	Emotion identification, emotion discrimination, and emotion matching.	The patient group performed significantly worse than control group on measures of cognition and emotional perception.
03	Dourado, 2019^56^	52 participants AD mild n=29 AD moderate n=23	Face drawings Sadness, happiness, anger, or surprise	Matching: instructions–match target face with one of four alternatives; point/verbal response to another example of same emotion and three other expressions. Selection: instructions–point to the sad faces/choose face that matches situation; point response to one of four alternatives.	The subjects must select the target of three other distractors in the same category. Identity discrimination: (same/different emotion). Indicate which of the four drawings best depicts that specific emotion. And indicate the drawing that best described the emotion he had inferred from the stimulus.	Emotional processing difficulties across AD stages. However, when participants needed to recognize the most salient emotion in a situation with evident emotional content, the results suggest that in both groups, there was no influence of cognitive impairment.
04	Duclos, 2018^42^	n=60 AD 20 HOS 20 HYS 20	The Peter and Mary emotion tasks Battery. Two basic emotions (i.e., anger and surprise), two self-consciousness emotions (i.e., embarrassment and pride), plus the neutral state.	(1) context task, (2) face task, and (3) context–face task	Identification of emotions (What is the emotion expressed by the character?), Discrimination of emotions (Which of the four situations could induce that emotion), Correspondence of emotions (Does the emotion expressed by the character correspond to the context?).	The results suggest that patients with AD have difficulty attributing emotional mental states, and deficits in social norm knowledge and the presence of incongruent information may heighten this difficulty.
05	Giffard, 2009^43^	AD 26 CG 26	Lists of words (neutral, positive, and negative).	Semantic priming task The lexical decision task.	Identification of words	The results show that this emotional process is preserved in AD and may even help patients to bind semantically close emotional concepts together more tightly.
06	Kalenzaga, 2013^44^	AD 22 CG 21	Two lists of 30 personality trait adjectives were selected from a normalized pool. Paradigms Remember/Know/Guess	Remember/Know/Guess.	The participants were asked to underline the words they recognized from the study list and to indicate whether or not they had a conscious recollection of the learning sequence.	Results indicate that self-reference increased autonoetic consciousness only for emotional and particularly negative trait adjectives.
07	Kalenzaga, 2013^45^	n=40 AD 22 CG 18	Two lists of 30 personality trait adjectives were selected from a normalized pool.	Remember/Know/Guess.	Read aloud, answer questions about yourself, and underline the words you recognized from the list of studies. Report whether or not they had a conscious memory of the learning sequence.	Results for patients with AD show that self-reference increased autonoetic consciousness only for emotional and particularly negative trait adjectives.
08	Kalenzaga, 2014^46^	n=33 AD 18 CG 15	64 nouns/words were divided into four lists of 16 words (i.e., 8 neutral, 4 positive, and 4 negative) matched for frequency, concreteness, and valence.	Remember/Know/Guess	Underline the identified words from the list of studies and indicate whether or not they had a conscious recall of the learning sequence.	Patients with AD were as able as normal controls to benefit from the emotional content of information to improve the recollection of details.
09	Kalenzaga, 2016^47^	n=155 YA 38 OA 39 VOA 37 AD 41	64 nouns/words were divided into four lists of 16 words (i.e., 8 neutral, 4 positive, and 4 negative) matched for frequency, concreteness, and valence.	The task was presented using the SuperLabPro software.	Participants had to read aloud and learn the words, had to generate a sentence containing the word, and after trying four items, the participants had to remember the four words and the corresponding phrases produced.	The results indicated that the positivity bias is most likely to occur in individuals whose cognitive functions are preserved, after long retention delay, and in experimental conditions that do not constrain encoding.
10	Laisney, 2013^48^	AD 16 CG 15	Preference judgment task, False-belief task, and Reading the Mind in the Eyes test	Reading the Mind in the Eyes test	Identification in Matching emotions and Discrimination	We observed impaired performances by patients with AD on all the ToM tasks. However, affective ToM seemed affected to a lesser degree than the cognitive dimension.
11	Maki, 2013^49^	n=54 AD 12 ANC 17 YNC 25	600 colored face images of 6 basic emotional expressions (i.e., happiness, surprise, anger, sadness, fear, and disgust). DB99.	The participants were required to answer by touching the 100% face that corresponded to the expression of intermediate face.	Emotion identification, (What is the emotion expressed by the face/photo on the computer screen?)	In recognition of happiness, there was no difference in sensitivity between YNC and ANC, and between ANC and patients with AD. Patients with AD were less sensitive than ANC in recognition of sadness, surprise, and anger.
12	Mograbi, 2012^50^	n=43 AD 22 CG 21	The groups watched four films, namely, one neutral, one positive, and two negative. Joyful and sad, relaxed and frightened, calm and irritated, and hopeful and hopeless. Joy, surprise, fear, sadness, disgust, anger, and contempt were evaluated.	The groups watched four film excerpts, namely, one neutral, one positive, and two negative.	Self-evaluation (How do you feel after watching this?’). For each emotion, participants were asked to rate how they were feeling.	The study indicates that patients show emotional reactions congruent with stimulus valence, but that subjective reactivity is reduced in comparison with healthy older adults for negative material
13	Mograbi, 2012^51^	AD 23 CG 21	Two success–failure computerized paradigms were developed, i.e., one based on reaction time and the other on memory. Frustrated, satisfied; disappointed, satisfied; ashamed, feeling good about himself; and bored, energetic.	Success condition task and one in a failure condition.	Performance evaluation	The results indicated that, relative to controls, patients with AD exhibited impaired awareness of performance, but comparable differential reactivity to failure relative to success tasks, both in terms of self-report and facial expressions.
14	Monti, 2010^52^	PRAD 19 HE 15 YA 22	Ekman photos: Happy and Fear	The nonemotional conflict task versus emotional conflict task.	Emotion identification, (What is the emotion expressed by the face/photo on the computer screen?)	It was found that, compared to the young adult cohort, the healthy elderly displayed deficits in task-set shielding in the non-emotional but not in the emotional task, whereas PRAD subjects displayed impaired performance in both tasks.
15	Sapey, 2015^53^	n=78 AD 39 CG 39	Photographs of faces and answer options were displayed on a computer screen. Happiness, anger, disgust, fear, and neutral.	Behavioral tasks, facial gender recognition task, and facial emotional expression recognition task.	Participants were asked to determine whether the face was more feminine or masculine. Participants were asked to select the label that best described the emotional expression	The results indicate that emotion recognition is impaired specifically, rather than as a consequence of global cognitive dysfunction.
16	Sava, 2017^54^	n=63 AD 17 HO 21 HY 25 n=60 AD 18 HO 21 HY 21	The stimuli were 96 black and white photographs (7 cm × 5 cm) with 32 human adult faces against a gray background (each one depicting sad, neutral, and happy emotional expressions). Stimuli were selected from the Montreal Set of Facial Displays of Emotion	Delayed matching-to-sample task, and Emotion classification task.	Identity discrimination: indicate who the same person is. Emotion identification (What is the emotion expressed by the character?)	Results suggest that the positivity effect in memory is not entirely due to the sense of familiarity for smiling faces.
17	Seidl, 2012^57^	47 participants AD	Photos-IAPS: joy, surprise joy, surprise, anger, fear, disgust, sadness, and contempt.	The participants were encouraged to focus their attention on each presented picture (“Let’s have a look at this one”); simultaneously, facial expression was videotaped.	Attentional focus in either emotion-eliciting or neutral images	The result that cognitive deficits are associated with loss of specific facial expression point toward a progressive loss of control of facial expressions, corroborating previous findings.
18	Torres, 2015^58^	AD 30 CG 30	Face drawings Sadness, happiness, anger, or surprise	Tasks of identifying faces, understanding facial emotions, recognizing expression of emotion, and understanding the nature of a situation and the appropriate emotional state that someone would experience in that situation	The subjects must select the target of three other distractors in the same category. Identity discrimination: (same/different emotion). Indicate which of the four drawings best depicts that specific emotion. And indicate the drawing that best described the emotion he had inferred from the stimulus	The findings suggest that people with mild AD have difficulties making emotional interpretations of the environment, mostly due to their worsening global cognition. People with AD had an impaired ability to perceive emotions from situations, particularly when the emotions presented were relatively subtle.
19	Werheid, 2011^55^	AD 18 CG 18	The stimulus material consisted of 192 portraits of Caucasian faces gathered from different databases and selected based on computer-assisted 7-step valence ratings: happy, neutral, and angry	The participants’ task was to decide whether they had previously seen a portrait of the depicted person. Finally, on completion of the task, subjects were asked to fill out the post-test PANAS forms	Identity discrimination: indicate whether same or different person	The pattern of results supports the view that the positivity-induced recognition bias represents a compensatory, gist-based memory process that is applied when item-based recognition fails.

AD: Alzheimer’s disease; ANC: aged normal controls; AS: antisaccades; CG: control group; FB: false belief; HE: healthy elderly; HO: healthy older; HOAs: healthy older adults; HOS: healthy older individuals; HY: healthy young; HYs: healthy young individuals; IAPS: International Affective Picture System; OA: older adults; OCs: older controls; PANAS: Positive and Negative Affect Schedule; PRAD: probable Alzheimer’s disease; PS: prosaccades; ToM: Theory of Mind; VOA: very old adults; YA: young adults; YNC: young normal controls.

### Results of assessment tasks

In eight studies,^39^ ^,^ ^48^ ^,^ ^49^ ^,^ ^52^ ^,^ ^53^ ^,^ ^54^ ^,^ ^55^ ^,^ ^57^ photos were used as stimuli, and the participants were invited to select the frame, which best described the emotional expression represented. No significant difference in performance was observed among healthy older adults and people with AD in two studies.^49^ ^,^ ^54^ The other six studies concluded that people with AD had worse performance than the control group, and only one study^52^ related the worse performance to the specific decline in emotion processing. Werheid et al.^55^ attributed the deficit to memory impairment in AD. The other four studies explained the worse performance of people with AD in more specific neurodegenerative processes in the amygdala and hippocampus and in the general progression of the disease.^39^ ^,^ ^48^ ^,^ ^53^ ^,^ ^57^

Dourado et al.^56^ and Torres et al.^58^ utilized the FACES protocol, which consists of four increasingly difficult facial recognition tasks. The first task investigated the visuoperceptual capacity of identifying faces. The second task examined the comprehension of facial emotions. The third task examined if people with AD can conceptually recognize the emotional expression, and the fourth, the most complex task, assessed the capacity of understanding the emotional nature of one situation. In the study of Dourado et al.,^56^ the results of task 1 have shown a relationship between the impairment of visuoperceptual capacity to identify faces and decreased performance related to cognitive competence. No differences between mild and moderate AD were observed in task 3. No clinical variables predicted the performance in task 4 among people with mild AD, even after the adjustment to the severity of cognitive decline; however, among people with moderate AD, there was a relationship between task 4 and the emotional recognition domain of awareness of the disease. In the baseline assessment of the study of Torres et al.,^58^ there was no relationship between tasks 1, 2, 3, or 4 and the clinical variables. No relation was observed between the global score of FACES and the sociodemographic or clinical variables. After 6 months, the study has shown a significant difference between both assessments on the scores of task 4. These results show that the emotional impairment in the mild stage of the disease is related to the cognitive impairment and with the progression of AD. However, the study had a small sample and a time span of only 6 months.

To determine the impact of emotion information in early attentional processes among people with AD, Bourgin et al.^41^ applied an eye-screening paradigm and a task that mixed prosaccades and antisaccades with the emotional stimulus. The results suggested that people with AD had difficulties in rapidly and automatically focusing their attention on the emotional characteristics of the faces.

Two studies^42^ ^,^ ^50^ used video clips as stimuli to assess the capacity of people with AD on the discrimination and tagging of emotions. Duclos et al.^42^ used the Peter and Mary test, which is composed of short videos with black-and-white images, without sound, and with two characters. This study also applied images with colorful drawings that represented several activities of daily living. The results demonstrated that people with AD had impairment in decoding emotional mental states of facial expressions.

Mograbi et al.^50^ applied movie excerpts, one neutral, one positive, and two negatives to investigate the influence among people with mild and moderate AD. The results showed that the participants demonstrated reduced reactivity to negative movies, but they exhibited a pattern of facial expressions similar to the control group in all other movie excerpts. Mograbi et al.^51^ investigated the emotional reactivity to success or failure among people with AD in comparison with a cognitive healthy control group. The study applied two experimental computerized paradigms of manipulation of success and failure, i.e., one based on reaction time and the other on memory. The results of the group of people with AD have shown a reduction in the awareness about the performance, with difficulties to discriminate between the success and failure conditions. In contrast, they have shown the same levels of emotional reactivity of the control group in both experiences.

Five studies^43^ ^,^ ^44^ ^,^ ^45^ ^,^ ^46^ ^,^ ^47^ used word lists classified according to the emotional valence as the stimulus. Kalenzaga et al.^47^ reported that people with AD have shown bias toward positive stimuli, especially in the recognition task after the semantic processing instruction, while, in the group of healthy older adults, the positive preference was observed in both recovering tasks. In the study of Kalenzaga et al.,^46^ both people with AD and cognitive healthy controls may have benefited from the emotional content of the information, with better performance in the retrieval of the information. People with AD were as able as normal controls to benefit from the emotional content of information to improve the recollection of details.

One study^44^ showed that although people with AD had an explicit view of themselves, they only pointed out negative characteristics. Another study^45^ showed that people with AD produced fewer correct answers when compared with cognitive healthy controls. The data confirm that, in AD, there is a specific impairment in autonoetic awareness and show that declarative memory impairment, which is the characteristic of AD pathology, is mainly an impairment in episodic memory. When a lexical decision task was applied^43^ by comparing the automatic semantic effects between people with AD and cognitive healthy control group, the results reveal that in both groups, there were primary semantic effects to neutral concepts, but they were decreased in comparison with those effects to emotional concepts, especially negative ones. These results have shown that the emotional processing is preserved in AD and can even help people with AD to reconnect to the emotional semantic concepts.

### Results of the emotional valence assessment tasks

The eight studies that assessed emotional valence (i.e., positive, negative, and neutral)^41^ ^,^ ^43^ ^,^ ^44^ ^,^ ^45^ ^,^ ^46^ ^,^ ^47^ ^,^ ^50^ ^,^ ^51^ had differing results. In the study by Bourgin et al.,^41^ people with AD did not show differences in relation to the emotional category in any of the tasks. These results suggest that the early emotional attention is impaired among people with AD.

The analysis of Kalenzaga et al.^46^ indicated that, compared with the control participants who have recognized both emotional and neutral words, people with AD recognized more neutral than emotional words. This result is in accordance with the studies that reported that people with AD did not retrieve emotional items better than they retrieve the neutral ones.^60^ ^,^ ^61^ ^,^ ^62^ In the study of Kalenzaga et al.,^47^ there was a significant effect of emotional valence, indicating that participants remembered more positive than neutral words. The results suggested that the positivity bias is presented only if the memory performance scores have a certain pattern.

In the study of Mograbi et al.,^51^ there were no significant differences among the groups in the positive and negative tasks. Therefore, the affective valence of the failure experience is processed even though there is no awareness about the task performance. In the experiment with movie excerpts, Mograbi et al.^50^ found that participants of the AD group may have impairments in self-reported reactivity to negative stimuli. All of the emotions and general reactivity were significant, with more positive emotional changes in response to the positive excerpt and negative alteration in the two negative excerpts.

Three studies have shown different results in comparison with the previous ones, which show a more significant effect of the positive emotional valence among people with AD and controls. Giffard et al.^43^ showed a facilitating effect of the supplementary emotional component in the semantic processing. The primary semantic effects were higher to the negative concepts than to the neutral and positive ones.

The study of Kalenzaga et al.^44^ indicated that, in comparison with other encodings, the negative valence is salient to the self-reference of people with AD. Previous studies that explore the effect of valence on emotional processing show that the information remembered about oneself is generally related to the positive valence.^63^ ^,^ ^64^

In the results of Kalenzaga et al.,^45^ while investigating the interaction between the decoding condition and the emotional valence, there was a contrasting pattern of performance. Only the negative valence had a significant effect on the autonomous awareness of people with AD in the condition of self-reference. However, the self-reference effect was also marginally significant to the adjectives of positive characteristics, and pairwise comparisons have shown that there was no significant difference between the answer rate to positive and negative adjectives.

### Findings of studies about cognitive impairment an emotion processing in Alzheimer’s disease

Seid et al.^57^ suggested that cognitive changes influence the recognition of facial expressions. The study of Bourgin et al.^41^ agreed with this point as it reported that people with AD had difficulties in quickly and automatically focusing their attention on the emotional features of faces. Other studies^39^ ^,^ ^42^ ^,^ ^48^ ^,^ ^52^ ^,^ ^53^ have also identified that cognitive impairment and disease severity are related to deficits in the recognition of facial expression, particularly in more complex tasks that supposedly would require more cognitive resources. In agreement, some studies^56^ ^,^ ^58^ suggest that difficulties in recognizing emotions are more prevalent in relation to more subtle emotions among individuals with AD.

However, the study of Werheid et al.^55^ suggested that the emotion-based aspects of old/new decisions for faces are preserved in people with mild AD. Gifard et al.^43^ and Sava et al.^54^ showed that emotional processing is preserved in AD and may even help people with AD to link semantically close emotional concepts more tightly. These results are in line with previous findings showing no difference in facial emotional expression processing between healthy older participants and participants of the AD group,^35^ ^,^ ^65^ especially for faces with happy expressions.^49^

The results shown by Kalenzaga et al.^46^ corroborate the findings of previous studies^44^ ^,^ ^45^ and confirm that people with AD have a specific deficit in the episodic component of memory for words. Furthermore, Kalenzaga et al.^47^ indicated that the positivity bias is most likely to occur in individuals whose cognitive functions are preserved, after a long retention delay, and in experimental conditions that do not constrain encoding.

Mograbi et al.^50^ showed that people with AD showed emotional reactions congruent with stimulus valence, but the subjective reactivity is reduced in comparison with healthy older adults for negative material. Mograbi et al.^51^ indicated a dissociation between impaired performance judgment and preserved emotional reactivity to failure in AD.

## DISCUSSION

To better understand the impairment in emotion processing in people with AD, this systematic review analyzed 19 studies. The main findings suggest the following: (1) people with AD may have impairments in the ability to process emotions, but there are controversies about the beginning of emotion perception impairment among them, (2) there is not a methodological standard in the assessment of emotion processing, which may influence the results and contribute to a variability and lack of consistency of findings in the area, (3) the studies disagree whether the impairment in emotion processing is related to global cognitive impairment or to a decrease in a more specific system of emotion processing, (4) the impairment in emotion processing may be independent of stimulus or task used in the testing of various emotional aspects, (5) there are discrepancies in identifying emotions; while some studies showed that the emotions with negative valence may be more impaired than the positive emotions, other studies reported that the degree of this impairment may be influenced by disease severity and level of excitement (e.g., anger elicits more excitement and is better perceived than sadness), and (6) the analysis of the effect of emotional valence has shown influence on cognitive performance among people with AD.

### The beginning of emotion processing impairment among people with Alzheimer’s disease

The beginning of emotion processing impairment among people with AD has not been discussed enough in any of the evaluated studies. There is almost a consensus that the abilities of emotion perception are frequently impaired even among people with mild dementia due to the impairment of limbic system.^66^ ^,^ ^67^ ^,^ ^68^ ^,^ ^69^ Therefore, the neurodegenerative process among people with AD may be responsible for increased impairment in emotion perception.^70^ ^,^ ^71^ However, some studies consider that the emotion processes are relatively preserved in mild dementia, in comparison with other cognitive domains, such as semantic memory.^35^ ^,^ ^37^ Other studies also support that people with AD may retain the capacity of understanding the emotion, independently of the decrease in the cognitive capacity, and they remain capable of being involved in nonverbal communication that may occur in their interpersonal relationships.^72^ Nonetheless, only the study reported by Giffard et al.^43^ suggested that emotion processing is relatively preserved in mild dementia, in comparison with other cognitive domains. Conversely, some studies^39^ ^,^ ^42^ ^,^ ^48^ ^,^ ^50^ ^,^ ^52^ ^,^ ^53^ ^,^ ^56^ ^,^ ^58^ point out that the impairment of emotion processing may start even in the mild cases of AD due to the cognitive impairment.

We may observe that the results of the reviewed studies highlight the beginning of the impairment of emotion processing in different stages of the disease. However, these discrepancies among studies may be a result of the variability of objectives and different study methodologies, which may influence and hinder the process of identifying the key points. In addition, the complexity of tasks suggests multiple steps/components that could result in failures/low scores, some of which may not be related to the core features of emotional processing. For example, failed naming of emotions based on face stimuli could be tied to emotion processing/recognition or language disruption in semantic networks. Therefore, more longitudinal studies are necessary to identify the exact stage of the disease in which those difficulties occur. Identifying earlier this kind of impairment would be crucial to a differential diagnosis and to the development of strategies of intervention to facilitate communication and emotional expression of people with AD.

### Performance in emotional processing in individuals with Alzheimer’s disease

Most studies used traditional methods (i.e., naming of emotions, discrimination, identification, and correspondence) that have been used in the last decade to assess emotion processing. Due to the cognitive competences involved in each of these different procedures, it is understood that the implicit cognitive processes involved in each task may be affected by the disease. Thus, we may highlight that there is not a consensus about the impairment involved in the capacity of decoding emotions among people with AD, as some results show that there are abilities preserved in AD, while others report deficits in this ability. Maybe, these controversies are caused by the variability of stimuli used in the assessment. This diversity may produce bias or inconsistency of results that are sometimes contradictory. For example, Laisney et al.^48^ reported that people with AD may have more difficulties in tasks that involve various cognitive domains, such as language, verbal working memory, and social cognition, in comparison with their difficulties in affective tasks. In contrast, Bourgin et al.^41^ suggest that the early emotional attention is impaired among people with AD in a technique of sight-screening during a simple cognitive task. Overall, the results have been generally attributed to the characteristic decline in AD. However, we may consider that the lack of standardization of assessment instruments to consistently investigate whether people with AD maintain their preserved capacity of recognition of others emotions as well as the lack of expression of their own emotions may be directly related to their performance.

### Capacity of perceiving emotions

In relation to specific emotions involved in the decoding tasks, some studies investigated the ability to identify specific emotions such as happiness or sadness. Previous findings^31^ ^,^ ^35^ ^,^ ^73^ ^,^ ^74^ highlight that the capacity to process some emotions is preserved in AD (e.g., happiness and disgust). Other studies highlight that the participants have particular impairment in the decoding of emotions, such as fear.^6^ ^,^ ^25^ ^,^ ^75^ There is contrasting evidence using a longitudinal design to assess the recognition of facial expressions among people with AD. A study^58^ reported that the participants continued to appropriately recognize the basic emotions represented in simple drawings but had more difficulties in a more complex task (i.e., identifying the emotional state that would be experimented in a particular situation). However, the results of Sapey-Triomphe et al.^53^ in a cross-sectional design showed that people with AD were impaired in the recognition of each one of the various basic emotions (i.e., anger, fear, happiness, and, on a lower degree, disgust). The decline in the ability of emotional decoding was significantly related in AD to all types of emotions. There are assumptions that the performance in the recognition of emotions is related to the volume of expected brain structures. However, the performance on the recognition of emotions was negatively related to the majority of volumes in the gray matter in the interest regions. In particular, the performance in recognition of fear was negatively related to the amygdala volume.^53^ These data contradict other studies^76^ ^,^ ^77^ that associated the amygdala function in the processing of fear. Various studies highlight the central role of the amygdala in the emotion processing^17^ ^,^ ^19^ ^,^ ^20^ ^,^ ^78^ and support the hypothesis that the amygdala is involved in the process of the response of the stimuli to several emotions, which may explain why the decline in the emotional coding of people with AD is not restricted to one specific emotion such as fear.^79^ It is reasonable to consider that the impact of the neurodegenerative process associated with AD may affect the identification of specific emotions. This point may indicate that regional involvement in each kind of emotion recognition follows distinct patterns, which supports the localizationist view that postulates unique regional networks for each emotion.^17^ Due to the progressive nature of AD, longitudinal studies with the support of neuroimaging could provide more comprehension of the change patterns in the process of discrimination of emotions among people with AD.

### Relationship between emotion processing and cognitive domains

The relationship between deficits in emotion processing and cognitive functioning in people with AD is still underestimated. Although decline in emotion perception may occur, the impact of the global cognitive decline associated with AD across studies varies. Some studies suggest that global cognitive performance affects the performance on tasks of emotion perception among people with AD, but other studies show a decline in the recognition of emotion, independently of the cognitive performance. We hypothesized that these results may be due to the considerable heterogeneity of methods and assessment of cognitive and emotional processes.

Few studies investigate the relationship between cognitive impairment, the severity of the disease, and the impact on the perception of emotions.^53^ ^,^ ^56^ ^,^ ^58^ Moreover, some authors support that emotion processing impairment exists independently of disease severity. Various tools have been used to assess cognitive functions among people with AD. However, most studies have applied the MMSE to assess the association between cognitive state and emotion processing in tasks and procedures. The emotion decoding performance in AD was significantly related to MMSE scores, but some studies did not find this significant relation. Therefore, the strength of these results may be cautiously considered since the MMSE is a tool for screening cognitive decline, susceptible to education and socioeconomic status. Studies that only include screening tests may have limited validity in their findings. A comprehensive neuropsychological assessment of different cognitive domains would have more power to disentangle, in which cognitive functions are more or less related to the emotional processing in AD. Thus, we may consider that the relationship between emotion processing and global cognitive capacity is not clear.

### The effect of different emotions in Alzheimer’s disease

The research about emotional valence and its impact on emotion and cognitive processing in AD has been explored, and the results are inconsistent. Some studies have shown the existence of a positive bias.^46^ ^,^ ^47^ ^,^ ^49^ ^,^ ^55^ For example, the study of Werheid et al.^55^ shows that the positive bias is really present and affects the performance of people with AD, as well as in cognitive healthy old adults. These data represent evidence of preservation of emotion processing in people with AD. However, we may highlight that the results of studies have shown significant difference among assessed groups, and, therefore, we may consider that, in general, there is a decline in the cognitive capacities of processing emotions and responding to them.

Our study has some limitations that should be considered. There are a limited number of studies that investigate emotional processing in AD, and many of them have low statistical power. In addition, the variety of objectives and methodologies in the reviewed studies hinder the identification of key points. More research is necessary, therefore, to establish the attributions of emotions in relation to cognition.

Our main target was to analyze the relationship between emotion processing and cognition in mild and moderate AD. We concluded that there is evidence that suggests the presence of emotion processing deficits even among people with mild AD. However, the relationship between emotion processing and cognition is not clear. The existing literature presents contradicting results and reports emotion processing impairment in relation to cognition but does not discuss the possible implications of this impairment in social (dys)function. Crucially, emotion processing impairment may negatively impact the well-being of people with AD and their caregivers, which could be an important discussion for future studies.

More longitudinal studies are necessary to investigate the relationship between cognition and emotion processing during the disease progression. Potentially confusing factors such as global cognitive decline, presence of neuropsychiatric symptoms, and mood must be observed. Studies that include neuroimaging data, such as volumetric analyses of the amygdala, may also enhance the comprehension of the processes involved in emotion recognition among people with AD. Therefore, the progressive neuropathological alterations that characterize the disease may impact on emotion processing, and a sensitive focus on this process may help to refine interventions that encompass social interaction.
